# Mitochondrial Ca^2+^ Uptake from Plasma Membrane Cav3.2 Protein Channels Contributes to Ischemic Toxicity in PC12 Cells*

**DOI:** 10.1074/jbc.M112.428128

**Published:** 2013-03-18

**Authors:** Yves Gouriou, Philippe Bijlenga, Nicolas Demaurex

**Affiliations:** From the Departments of ‡ Cell Physiology and Metabolism,; § Fundamental Neuroscience, and; ¶ Clinical Neurosciences, University of Geneva, Geneva CH-1211, Switzerland

**Keywords:** Calcium Signaling, Ion Channels, Ischemia, Mitochondria, Stroke

## Abstract

T-type Ca^2+^ channel inhibitors protect hippocampal CA1 neurons from delayed death after global ischemia in rats, suggesting that Cav3.1, Cav3.2, or Cav3.3 channels generate cytotoxic Ca^2+^ elevations during anoxia. To test this hypothesis, we measured the Ca^2+^ concentration changes evoked by oxygen and glucose deprivation (OGD) in the cytosol and in the mitochondria of PC12 cells. OGD evoked long-lasting cytosolic Ca^2+^ elevations that were reduced by Cav3.2 inhibition (50 μm Ni^2+^) and Cav3.1/Cav3.2 silencing and potentiated by Cav3.2 overexpression. The kinetics of the sustained cytosolic Ca^2+^ elevations occurring during OGD directly correlated to the extent of cell death measured 20 h after reoxygenation, which was decreased by Ni^2+^ and Cav3.1/Cav3.2 silencing and increased by Cav3.2 overexpression. Ni^2+^ and Cav3.1/Cav3.2 silencing delayed the decline of cellular ATP during OGD, consistent with a reduction in the Ca^2+^ load actively extruded by plasma membrane Ca^2+^ pumps. The cytosolic Ca^2+^ elevations were paralleled by mitochondrial Ca^2+^ elevations that were also increased by Cav3.2 overexpression and decreased by Ni^2+^ but not by Cav3.1/Cav3.2 silencing. Overexpression and silencing of the mitochondrial Ca^2+^ uniporter, the major mitochondrial Ca^2+^ uptake protein, revealed that the cytotoxicity was correlated to the amplitude of the mitochondrial, rather than the cytosolic, Ca^2+^ elevations. Selective activation of T-type Ca^2+^ channels evoked both cytosolic and mitochondrial Ca^2+^ elevations, but only the mitochondrial responses were reduced by Cav3.1/Cav3.2 silencing. We conclude that the opening of Cav3.2 channels during ischemia contribute to the entry of Ca^2+^ ions that are transmitted to mitochondria, resulting in a deleterious mitochondrial Ca^2+^ overload.

## Teaser

**Background:** T-type Ca^2+^ (Cav3.2) channel inhibitors prevent neuronal death during ischemia, but the cytotoxic signals have not been resolved.

**Results:** Cav3.2 channels generate cytotoxic Ca^2+^ elevations in the cytosol and mitochondria of PC12 cells deprived of oxygen and glucose.

**Conclusion:** Ca^2+^ transfer from Cav3.2 channels to mitochondria contributes to ischemic toxicity.

**Significance:** Cav3.2 channels and the mitochondrial Ca^2+^ uniporter (MCU) are potential targets for the treatment of stroke.

## Introduction

The brain is one of the most energy-consuming organs, and disturbances in cerebral blood flow that deprive brain regions of oxygen and glucose rapidly cause metabolic defects by preventing the synthesis of the cytosolic ATP required for normal neuronal function. Within minutes of ischemia, neurons lose the ability to maintain ionic gradients, depolarize, and their cytosolic Ca^2+^ concentration ([Ca^2+^]_cyt_)^2^ increases sufficiently to trigger the exocytosis of excitatory neurotransmitters, leading to a secondary massive entry of Ca^2+^ that extends to nearby neurons in a deadly cascade termed excitotoxicity. Increased Ca^2+^ release from the endoplasmic reticulum (ER), increased Ca^2+^ influx across plasma membrane (PM) channels, and decreased Ca^2+^ extrusion have all been implicated in the pathogenesis of the [Ca^2+^]_cyt_ overload during ischemia (1). Cleavage of the Na^+^/Ca^2+^ exchanger by calpain was shown to prevent Ca^2+^ extrusion, potentiate Ca^2+^ overload (2), and reverse mode operation of the Na^+^/Ca^2+^ exchanger to contribute directly to Ca^2+^ entry, driven by increased cytosolic sodium build-up during ischemia (3) (reviewed in Ref. 4). Ionotropic glutamate receptors are recognized as the major Ca^2+^ entry pathway that mediates excitotoxicity (5) (reviewed in Ref. 1). In addition, during both the ischemic and reperfusion phases, Ca^2+^ entry is mediated by voltage-gated Ca^2+^ channels as well as acid-sensing ion channels (6) and Ca^2+^-permeable transient receptor potential channels (7, 8), which are activated by the acidosis and the high concentrations of reactive oxygen species that develop around ischemic neurons (9). Dihydropyridine-sensitive L-type voltage-dependent Ca^2+^ channels (Cav1.2) were long considered to be the main non-excitotoxic Ca^2+^ entry pathway, as Cav1.2 opening upon neuronal depolarization can cause massive Ca^2+^ entry during ischemia. However, clinical studies showed little benefit of dihydropyridine analogues in acute ischemic stroke patients (10). Inhibition of the lower capacity T-type voltage-gated Ca^2+^ channels (Cav3.1 and Cav3.2), on the other hand, was shown to protect rat organotypic hippocampal slice cultures from ischemia-induced delayed cell death (11) and to protect CA1 neurons from delayed death after global ischemia in rats (12). T-type Ca^2+^ channels can remain open for long durations at voltages close to the resting potential, generating a “window current” that allows a small but sustained entry of Ca^2+^ ions that can drive long-lasting processes such as myoblast differentiation (13). Such a small and sustained entry of Ca^2+^ could initiate a Ca^2+^-dependent apoptotic or necrotic cascade during ischemia (14), but no direct evidence that T-type channels contribute to pathological Ca^2+^ signals has been provided to date.

Mitochondria are intracellular signaling hubs that link Ca^2+^ homeostasis to cell metabolism and fate by their ability to sequester Ca^2+^ ions (15). The negative potential of the mitochondrial matrix drives the influx of Ca^2+^ ions across a low-affinity mitochondrial Ca^2+^ uniporter (MCU) that was identified recently (16, 17). MCU-mediated Ca^2+^ uptake allows mitochondria to act as mobile Ca^2+^ buffers that mitigate [Ca^2+^]_cyt_ elevations and prevent the Ca^2+^-dependent activation or inactivation of PM channels (reviewed in Ref. 18) and to translate [Ca^2+^]_cyt_ changes into metabolic responses (19). Moderate elevations in the mitochondrial matrix free Ca^2+^ concentration ([Ca^2+^]_mit_) activate enzymes of the tricarboxylic acid cycle to boost ATP production (20), whereas excessive [Ca^2+^]_mit_ elevations can activate the mitochondrial permeability transition pore, increasing the permeability of the inner mitochondrial membrane and triggering the release of pro-apoptotic factors that initiate the cell death cascade (reviewed in Ref. 21). Accordingly, MCU-overexpressing cells were more sensitive to apoptosis triggered by ceramide and hydrogen peroxide (17). The paradox that mitochondrial Ca^2+^ uptake requires high micromolar Ca^2+^ concentrations never achieved in the bulk cytoplasm of living cells (22) has been resolved by the discovery that mitochondria can be exposed to transient microdomains of high [Ca^2+^]_cyt_ during physiological and pathological Ca^2+^ elevations (23, 24). Mitochondria can take up Ca^2+^ ions flowing across Ca^2+^ release channels on the ER (25) and voltage-gated Ca^2+^ entry channels on the PM (26) but not across store-operated Ca^2+^ entry channels (27), which are activated by electrostatic interactions with proteins on apposed cortical ER structures (28), which precludes mitochondria access. Whether mitochondria can take up Ca^2+^ ions flowing across Ca^2+^ entry channels during ischemia is not known.

To fill this gap of knowledge, we used genetically encoded fluorescent biosensors to measure the [Ca^2+^]_cyt_ and [Ca^2+^]_mit_ changes occurring during ischemia in PC12 cells and assessed the contribution of T-type Ca^2+^ channels and of the MCU by gene silencing and overexpression. PC12 cells express oxygen-sensitive potassium channels that were shown to regulate membrane depolarization during hypoxia (29) and express both high- and low-threshold voltage-gated Ca^2+^ channels (30), the predominant T-type Ca^2+^ channel isoform Cav3.2 being up-regulated by hypoxia-inducible transcription factors and recruited to the PM after exposure to chronic hypoxia (31). Our data indicate that both Cav3.2 and the MCU contribute to ischemic toxicity by allowing the sustained transfer of Ca^2+^ to subplasmalemmal mitochondria during anoxia.

## EXPERIMENTAL PROCEDURES

### 

#### 

##### Reagents

RPMI 1640, Lipofectamine 2000, penicillin, and streptomycin were obtained from Invitrogen. Fetal calf serum and horse serum were obtained from Brunschwig. Sodium dithionite and staurosporine were obtained from Sigma-Aldrich. The D3cpv and 4mtD3cpv constructs were provided by Drs. Laurent Combettes (Université of Paris-Sud) and Roger Tsien (University of California, San Diego, CA). Cytosolic FRET-based ATP indicators (ATeam, adenosine 5′-triphosphate indicator on the basis of the ∈ subunit for analytical measurements) were provided by Drs. Hiromi Imamura (Japan Science and Technology Agency, Tokyo) and Hiroyuki Noji (Osaka University).

##### Cell Culture, Transfection, and RNA Interference

PC12 cells were grown in RPMI 1640 l-glutamine medium containing 5% fetal calf serum, 10% horse serum, and 100 units/ml of penicillin and streptomycin. Cells were cultured in 95% air and 5% CO_2_ at 37 °C and subcultured every 7 days at a 1:10 dilution. Cells were plated on poly-L-lysine-coated coverslips or plates and grown for 24 h before transfection with siRNA and/or fluorescent proteins using Lipofectamine 2000. Experiments were performed 48 h after transfection. Cav3.1 and Cav3.2 silencing was achieved with a pool of three target-specific siRNAs (Santa Cruz Biotechnology, Inc., catalog nos. sc-61869 and sc-61870), and knockdown efficiency was verified by quantitative PCR.

##### Mitochondrial Isolation, Cell Lysis, and Western Blotting

Mitochondria were isolated by differential centrifugation in isolation buffer (300 mm sucrose, 10 mm HEPES, 0.5 mm EGTA (pH 7.6)) and lysed for 20 min on ice in lysis buffer (25 mm Tris-HCl (pH 7.6), 150 mm NaCl, 1% Nonidet P-40, 1% sodium deoxycholate, 0.1% SDS) supplemented with protease inhibitors (Roche) as described in Ref. 32. The lysate was centrifuged at 14,000 × *g* for 20 min, and the protein content of the supernatant was determined using the Bradford protein assay (Bio-Rad). 30 μg of total protein/lane was loaded on an SDS-PAGE gel. For immunoblotting, proteins were transferred onto a nitrocellulose membrane and probed with 1/500 anti-CCDC109A/MCU and anti-Tom20 (Santa Cruz Biotechnology, Inc., catalog nos. sc-246071 and sc-11415, respectively). Horseradish peroxidase-conjugated secondary antibodies 1/20,000 (Amersham Biosciences) were used, followed by detection by chemiluminescence.

##### Oxygen-Glucose Deprivation (OGD) Experiments

Cells were washed twice in a HEPES-buffered solution containing 140 mm NaCl, 5 mm KCl, 1 mm MgCl_2_, 2 mm CaCl_2_, 20 mm HEPES (pH 7.4) with NaOH at 37 °C, placed into an anoxic chamber (Miniature Incubator, Bioscience Tools), and flushed continuously with a mixture of 95% N_2_/5% CO_2_ at 37 °C (TC2-80-150, Bioscience Tools). A similar solution containing 2 mm of sodium dithionite and equilibrated with the N_2_/CO_2_ mixture for 5 min was injected into the chamber to initiate the OGD. Oxygen levels were monitored with a titanium-coated probe and a Neo Fox 1 channel oxy monitor system (Instech Laboratories, Inc.). After the OGD period, cells were washed twice with RPMI 1640 medium and cultured for 24 h in a normoxic environment. Control (non-ischemic) cells were exposed to a HEPES-buffered solution containing 10 mm glucose in an incubator with 95% air and 5% CO_2_ at 37 °C.

##### [Ca^2+^]_cyt_, [Ca^2+^]_mit_, and [ATP]_cyt_ Imaging

Cells were imaged on an Axiovert s100 TV microscope using a ×40, 1.3 numerical aperture oil immersion objective (Carl Zeiss AG, Feldbach, Switzerland) and a cooled, 16-bit charge-coupled device back-illuminated frame transfer MicroMax camera (Roper Scientific, Trenton, NJ). [Ca^2+^]_cyt_ and [Ca^2+^]_mit_ were measured with D3cpv and 4mtD3cpv, respectively, and cytosolic [ATP] was measured with ATeamcyto, all FRET-based indicators. Cells were excited at 430 nm through a 455DRLP dichroic filter and alternately imaged with 480AF30 and 535DF25 emission filters (Omega Optical). Image pairs were acquired every 10 s. Fluorescence ratios were calculated in MetaFluor 6.3 (Universal Imaging) and analyzed in Excel (Microsoft) and GraphPad Prism 4 (GraphPad). Ca^2+^ slopes were determined by a linear fit to the steepest part of the Ca^2+^ increase, recorded between 20 and 60 min after OGD initiation.

##### Cytotoxicity Measurements

Lactate dehydrogenase (LDH) release was measured 20 h after OGD with a cytotoxicity kit assay (Abcam) on the basis of the cleavage of a tetrazolium salt by LDH. 100 μl of culture medium was collected, centrifuged to remove cellular debris, and transferred to a 96-well plate. 100 μl of the reaction mixture was added to each well and incubated for 30 min at room temperature. Absorbance was measured at 490 nm, and LDH release was expressed as a percentage of total LDH activity, measured by lysing cells with Triton.

##### RNA Extraction and Real-time PCR

Two days after cotransfection, cells were harvested by trypsination, washed twice with phosphate-buffered saline, and subjected to cytofluorometric analysis. GFP-positive cells were sorted using FACStar+ (BD Biosciences). Total RNA was isolated from sorted cells using the NucleoSpin RNA II kit (Macherey-Nagel, Düren, Germany), and 0.5 μg of DNase-treated RNA was used to synthesize cDNA using a QuantiTec reverse transcription kit (Qiagen, Hombrechtikon, Switzerland). For each PCR reaction, 1/20th of the cDNA template was amplified in a 7900HT SDS system using Power SYBR Green PCR master mix (Applied Biosystems, Foster City, CA). Raw threshold-cycle (Ct) values obtained from triplicate PCR reactions were averaged and normalized to four endogenous control genes (egf1, GAPDH, rps29, and tubb). Primers used were as follows: rat Cav3.2, GGCGTGGTGGTGGAGA-ACTT/GATGATGGTGGGATTGAT; rat Cav3.1, ACCTGCCTGACACTCTGCAG/GCTGGCCTCAGCGCAGTCGG; and rat MCU, AGACTTTTTGG-CTCCTTTATGGAAA/GAATATGTTTATCCTGAAGCCAGAGA.

##### Immunofluorescence

Cells were fixed with 4% PAF for 20 min at 4 °C, permeabilized with 0.2% Triton+ 1% BSA for 20 min at 20 °C, incubated overnight at 4 °C with the primary antibody at 1/100 (sc-16263, Santa Cruz), washed and incubated for 30 min at 20 °C with the secondary antibody at 1/1000 (Alexa Anti-Goat 405 nm, Invitrogen), and mounted on glass slides with Dako mounting medium. Images were taken on a Confocal Laser Scanning Microscope (Zeiss CLSM 700) using a Plan-Apochromat 63x/1.4 Oil objective.

##### Electrophysiology

Currents were recorded in the whole-cell configuration of the patch-clamp technique with an Axopatch 200B amplifier and Clampex 10.3 software (Molecular Devices). Cells were plated 3 days before the recordings, performed at 20 °C. Borosilicate glass pipettes (World Precision Instruments) were pulled with a Sutter puller, fire polished, and had a resistance between 2 and 3 mΩ. The external solution contained 130 mm NMDG, 10 mm BaCl_2_, 1 mm MgCl_2_, 10 mm HEPES, 10 mm glucose, pH adjusted to 7.4 with HCl (278 mOsM/kg). The pipette solution contained 140 mm CsAsp, 10 mm HEPES, 1 mm MgCl_2_, 10 mm EGTA, pH adjusted to 7.4 with CsOH (277 mOsM/kg). 350 ms pulses to +0mV were applied from a holding potential of −80mV to evoke T-type Ca^2+^ currents. Data were filtered at 5 kHz, digitized at 50 kHz, and currents normalized to the cell capacitance (10–20 pF).

##### Statistical analysis

The significance of differences between means was established using the Student's *t* test for unpaired samples (*, *p* < 0.05; **, *p* < 0.01; ***, *p* < 0.001).

## RESULTS

**FIGURE 1.**
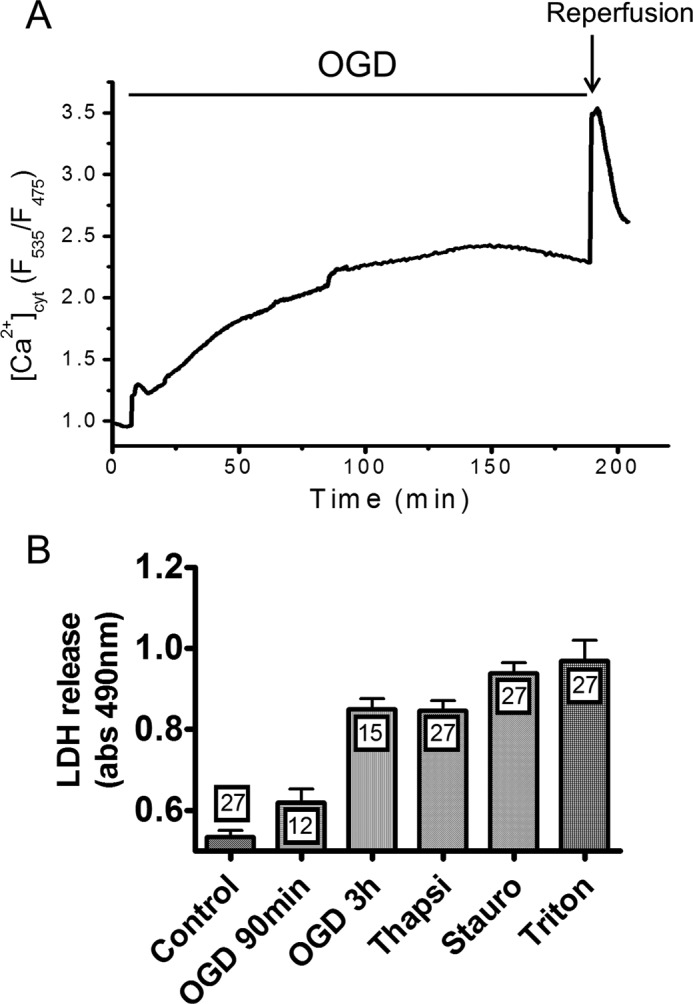
**[Ca^2+^]_cyt_ elevations and cytotoxicity evoked by OGD.** [Ca^2+^]_cyt_ was measured with the Cameleon indicator D3cpv, and cytotoxicity was measured by quantifying LDH release 20 h after anoxia. *A*, typical recording illustrating long-lasting [Ca^2+^]_cyt_ elevations evoked by OGD. OGD triggered a rapid [Ca^2+^]_cyt_ increase followed by a sustained elevation that reached a steady state after 2 h, and a further [Ca^2+^]_cyt_ increase was observed upon oxygen/glucose readmission. *B*, LDH release evoked by 90 min and 3 h of OGD and release evoked by thapsigargin (*Thapsi*) and staurosporine (*Stauro*), used as positive controls. Data are mean ± S.E. *n* is indicated inside the *bars*.

**FIGURE 2.**
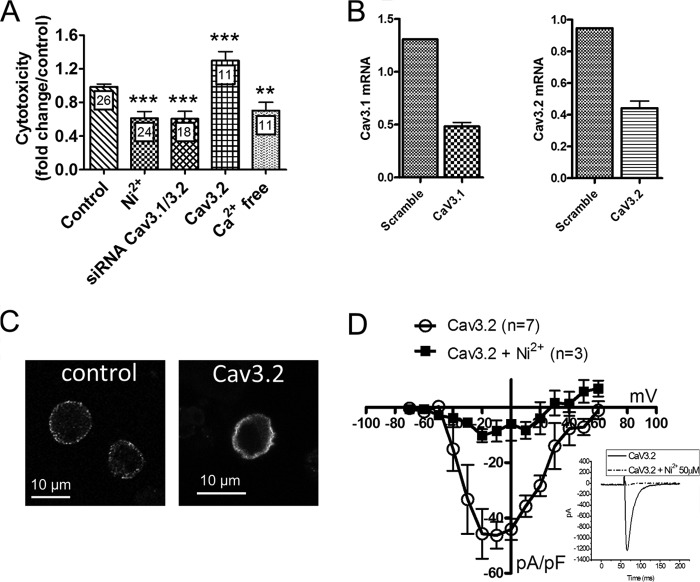
**Effect of T-type Ca^2+^ channels modulation on the cytotoxicity evoked by OGD.** *A*, effect of T-type channels modulation on the cytotoxicity evoked by OGD. Data are mean ± S.E., expressed as fold changes from the control condition. *n* is indicated inside the *bars. B*, Cav3.1 and Cav3.2 mRNA levels in cells treated with the indicated siRNAs, relative to house-keeping genes. *C*, Cav3.2 immunostaining in naïve PC12 cells (*left panel*) and in a cell overexpressing Cav3.2. *D*, current-voltage relationship of the peak inward current recorded before (○) and after (■) the addition of 50 μm Ni^2+^ in cells overexpressing Cav3.2. The *inset* shows the inward currents evoked by a 350-ms depolarization step to +0 mV in the absence and presence of 50 μm Ni^2+^. Data are mean ± S.E. from three to seven recordings. **, *p* < 0.01; ***, *p* < 0.001 (Student's *t* test for unpaired samples).

To study the impact of oxygen and glucose deprivation (OGD) on cytosolic Ca^2+^ levels we took advantage of genetically encoded indicators that, unlike synthetic dyes, allow long-term recordings when expressed in cells or tissues. PC12 cells were transiently transfected with the cytosolic FRET-based “Cameleon” Ca^2+^ indicator D3cpv, and [Ca^2+^]_cyt_ was measured by fluorescence microscopy during a 3-h long OGD generated by replacing the saline solution with a glucose-free medium bubbled with 95% nitrogen and 5% CO_2_. OGD triggered an immediate [Ca^2+^]_cyt_ increase followed within 20 min by a monotonic elevation that reached a steady-state plateau after 2 h (Fig. 1*A*). Subsequent oxygen/glucose readmission evoked a further, but transient, [Ca^2+^]_cyt_ increase. This procedure was associated with ∼80% cytotoxicity, as determined by measuring LDH release 20 h after the insult (Fig. 1*B*). A 3-h OGD was as efficient as thapsigargin in evoking LDH release and slightly less efficient than staurosporine, two commonly used cytotoxic agents (Fig. 1*B*). Depriving cells of oxygen and glucose for 90 min caused ∼25% cytotoxicity, whereas 30 min of OGD did not promote LDH release (not shown). Consistent with a causal role for Ca^2+^ entry in this cell death, cytotoxicity decreased by 30% when OGD was performed in the absence of extracellular Ca^2+^ (Fig. 2*A*). Strikingly, a similar protection was observed when OGD was performed in the presence of 50 μm Ni^2+^ (Fig. 2*A*), a concentration that selectively inhibits Cav3.2 channels (half maximal inhibition, 13 μm for Cav3.2 *versus* 250 μm and 213 μm for Cav3.1 and Cav3.3, respectively (33)). To confirm the implication of T-type Ca^2+^ channels in OGD-induced cell death, we knocked down the two T-type channel isoforms expressed in PC12 cells, Cav3.1 and Cav3.2 (31), using a combination of siRNAs that decreased their mRNA levels by 60–70% (Fig. 2*B*). Knockdown of T-type channels reduced cytotoxicity by 40% to the level observed in the absence of external Ca^2+^ or in the presence of Ni^2+^ (Fig. 2*A*). Concordantly, overexpression of the major endogenous T-type channel isoform of PC12 cells Cav3.2 (31) increased cytotoxicity by ∼30% (Fig. 2*A*). Transfection of cells with scrambled siRNA or with an empty vector did not alter OGD-evoked cytotoxicity (not shown). Functional overexpression was verified by immunofluorescence (Fig. 2*C*) and by recordings of transient inward currents activated by depolarizing voltage steps above −50 mV that were of large amplitude and inhibited by Ni^2+^ (*D*). These data indicate that, in PC12 cells, T-type calcium channels contribute to the cytotoxicity resulting from OGD.

**FIGURE 3.**
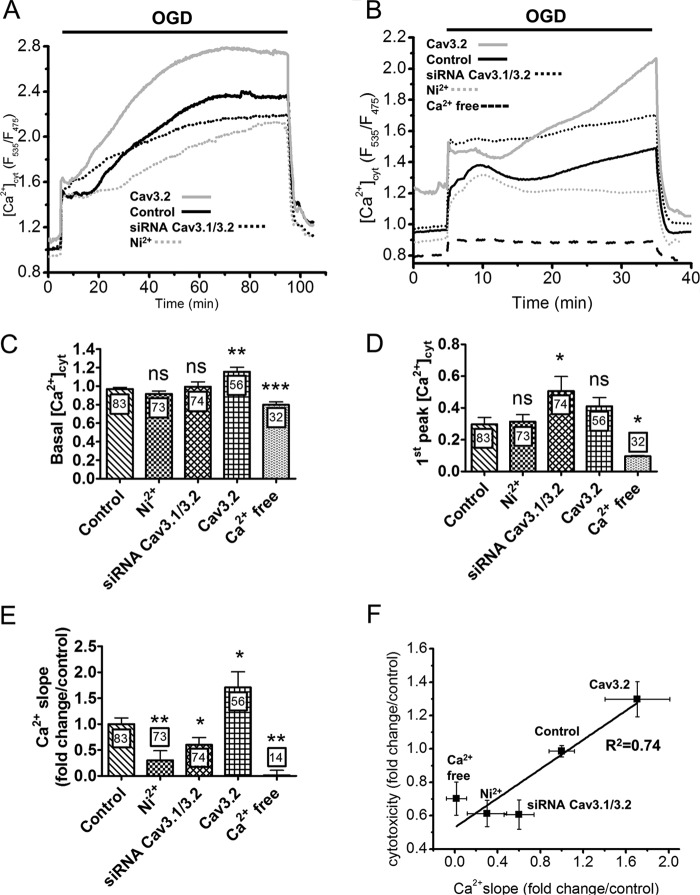
**Effect of T-type Ca^2+^ channels modulation on the [Ca^2+^]_cyt_ elevations evoked by OGD.** *A*, [Ca^2+^]_cyt_ recordings during 90 min of OGD. The delayed [Ca^2+^]_cyt_ elevation was reduced by Ni^2+^ and Cav3.1/Cav3.2 silencing and increased by Cav3.2 overexpression. Traces are the averaged [Ca^2+^]_cyt_ responses measured in the indicated conditions. *B*, [Ca^2+^]_cyt_ elevations evoked by 30 min of OGD. External Ca^2+^ removal prevented the elevations. *C*, basal [Ca^2+^]_cyt_ levels recorded under the different conditions. *D*, amplitude of the initial [Ca^2+^]_cyt_ elevation evoked by OGD. Data are mean ± S.E. from six to eight independent recordings. The total number of measured cells is indicated inside the *bars. E*, effect of T-type Ca^2+^ channel modulation on the slope of the secondary [Ca^2+^]_cyt_ elevation. Data are mean ± S.E. from three to four independent recordings, expressed as fold changes from the control condition. The total number of measured cells is indicated inside the *bars. F*, cytotoxicity as a function of the Ca^2+^ slope measured in the indicated conditions. Data are from Figs. 2*A* and 3*E. ns*, non-significant; *, *p* < 0.05; **, *p* < 0.01; ***, *p* < 0.001 (Student's *t* test for unpaired samples).

To confirm this link, we studied the impact of pharmacological and genetic manipulations of T-type Ca^2+^ channels on the cytosolic [Ca^2+^]_cyt_ elevations evoked by OGD. Shorter (30- and 90-min) OGD was applied to streamline the acquisition process because the prototypical biphasic [Ca^2+^]_cyt_ elevations could be well resolved during these time frames (Fig. 3, *A* and *B*). Notably, following 90 min of OGD, [Ca^2+^]_cyt_ did not increase further upon oxygen/glucose readmission but rapidly returned to basal levels (Fig. 3*A*). Predictably, [Ca^2+^]_cyt_ elevations were nearly abolished during OGD performed in the absence of external Ca^2+^, a procedure that also decreased the resting [Ca^2+^]_cyt_ concentration (Fig. 3, *B–D*). Ni^2+^, on the other hand, appeared to selectively impact the delayed phase of the [Ca^2+^]_cyt_ elevation, as it decreased the rates of the secondary [Ca^2+^]_cyt_ increase by 70% (Fig. 3, *A* and *E*) without altering resting [Ca^2+^]_cyt_ levels or the amplitude of the initial [Ca^2+^]_cyt_ peak (*C* and *D*). T-type Ca^2+^ channel knockdown also decreased the slope by 40% without altering resting [Ca^2+^]_cyt_ levels and, in addition, slightly increased the initial peak amplitude (Fig. 3, *C*–E). Conversely, Cav3.2 overexpression increased [Ca^2+^]_cyt_ levels at all times during OGD, increasing the rates of the secondary [Ca^2+^]_cyt_ elevation by 70%. These data indicate that T-type Ca^2+^ channels contribute to Ca^2+^ entry during the sustained phase of the [Ca^2+^]_cyt_ elevations evoked by OGD. The kinetics of the delayed [Ca^2+^]_cyt_ elevations measured during 30 min and 90 min of OGD correlated with the expression levels of T type Ca^2+^ channel isoforms and with cytotoxicity (Fig. 3*F*), a correlation that was particularly apparent for Ca^2+^ slopes measured during 90 min of OGD (R^2^ = 0.97). This tight relationship suggests that the Ca^2+^ ions entering across T-type channels during the delayed phase of the [Ca^2+^]_cyt_ elevations evoked by OGD are particularly toxic for cells.

**FIGURE 4.**
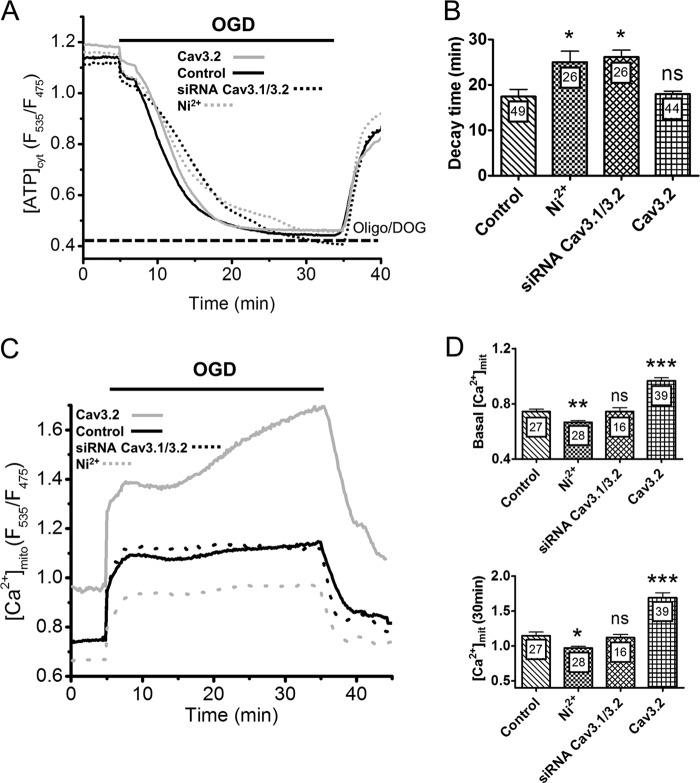
**Effect of T-type channels activity on cytosolic ATP levels and on [Ca^2+^]_mit_ during OGD.** *A*, averaged ATeam ratio fluorescence recordings during OGD. [ATP]_cyt_ levels decreased monotonically to reach a minimal value within 15 min and increased immediately upon reoxygenation. The *dotted line* indicates the level measured in cells exposed to 10 μg/ml oligomycin and 10 mm 2-deoxyglucose. *B*, averaged decay time in [ATP]_cyt_ levels during OGD. *C*, averaged [Ca^2+^]_mit_ recordings from cells exposed to 30 min of OGD. Ni^2+^ decreased [Ca^2+^]_mit_ at rest and throughout the OGD, whereas Cav3.2 overexpression increased resting [Ca^2+^]_mit_ and caused a delayed [Ca^2+^]_mit_ elevation during OGD. *D*, effect of T-type channels activity on resting [Ca^2+^]_mit_ levels (*top panel*) and on [Ca^2+^]_mit_ levels at the end of the 30-min OGD. Data are mean ± S.E. from three to five independent recordings. The total number of measured cells is indicated inside the *bars. ns*, non-significant; *, *p* < 0.05; **, *p* < 0.01; ***, *p* < 0.001 (Student's *t* test for unpaired samples).

During ischemia, mitochondrial and glycolytic ATP production is prevented, and the residual ATP is rapidly consumed by the activity of plasma membrane pumps that attempt to maintain a normal membrane potential and [Ca^2+^]_cyt_. Reducing the [Ca^2+^]_cyt_ elevation associated with OGD should, therefore, decrease ATP consumption by plasma membrane Ca^2+^ pumps and delay ATP depletion during ODG. To test this hypothesis we measured cytosolic ATP levels with the genetically encoded indicators ATeam (34). As expected, cytosolic ATP levels rapidly decreased upon OGD to reach a minimum value within 20 min, and recovered immediately when oxygen and glucose were subsequently restored (Fig. 4*A*). The values recorded after 20 min of OGD approached the levels measured in cells exposed to 10 μg/ml oligomycin and 10 mm 2-deoxyglucose (Fig. 4*A*, dotted line), indicating that cytosolic ATP contents were fully depleted. Interestingly, pharmacological and genetic invalidation of T-type channel activity markedly delayed the kinetics of ATP decrease, increasing the time required to reach the minimal ATP levels during OGD by ∼50% (Fig. 4*A* and *B*). Ca^2+^ removal had an even more pronounced delaying effect (not shown) whereas overexpression of Cav3.2 channels had no significant effect (Fig. 4*A* and *B*). None of these treatments had any effect on basal ATP levels (not shown), indicating that T-type Ca^2+^ channel inhibition specifically decreases ATP consumption during OGD. This ATP sparing effect might contribute to the reduced cytotoxicity observed in these conditions.

We next investigated whether T-type Ca^2+^ channels also contribute to mitochondrial matrix Ca^2+^ ([Ca^2+^]_mit_) elevations during OGD, using the mitochondria-targeted Cameleon probe 4mtD3cpv. The [Ca^2+^]_mit_ responses evoked by OGD mimicked the responses observed in the cytosol, with a rapid increase followed by a sustained plateau and, after short OGD, a rapid return to baseline levels (Fig. 4*C*). As observed in the cytosol, Cav3.2 overexpression increased the amplitude of [Ca^2+^]_mit_ elevations by accelerating the second phase of the response, whereas Ni^2+^ had the opposite effect (Fig. 4*D*). In contrast with the cytosolic data however, Cav3.2 overexpression increased, whereasNi^2+^ decreased, resting [Ca^2+^]_mit_ levels, whereas Cav3.1 and Cav3.2 silencing had no measurable impact on basal [Ca^2+^]_mit_ levels and on the [Ca^2+^]_mit_ elevations evoked by OGD (Fig. 4*D*).

**FIGURE 5.**
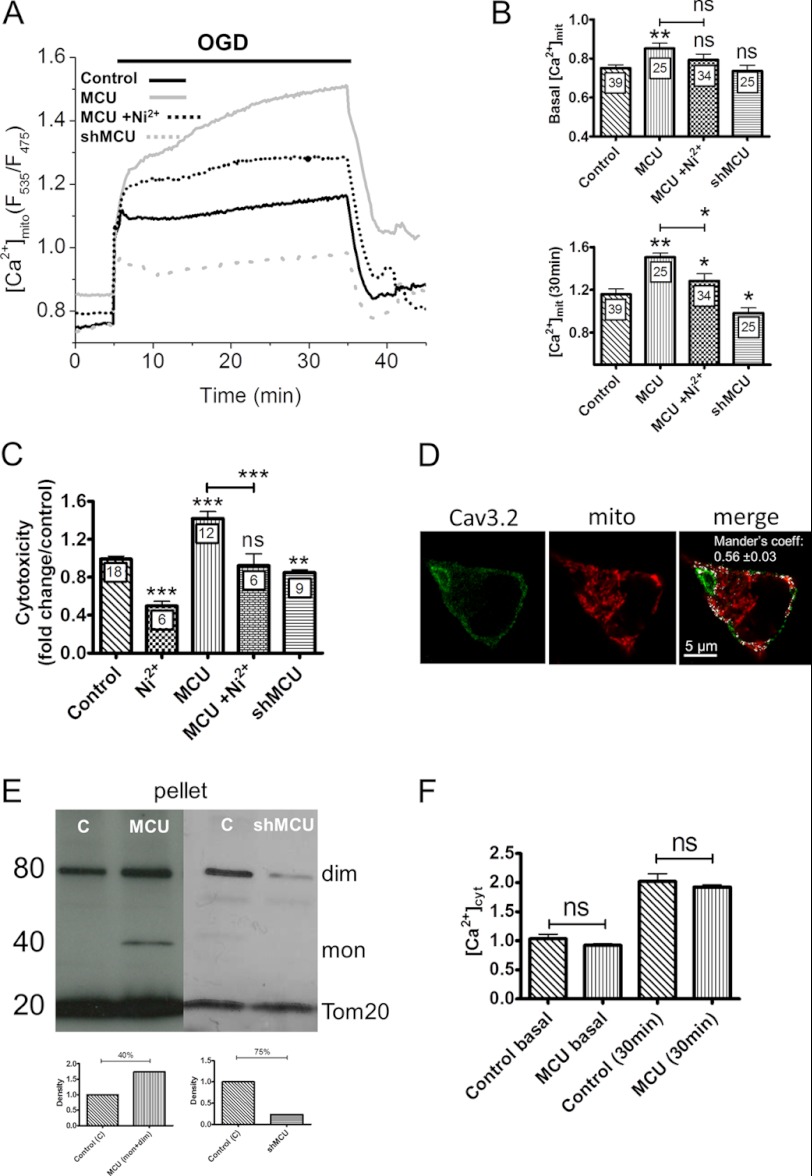
**Effect of MCU overexpression of [Ca^2+^]_mit_ elevations and cytotoxicity evoked by OGD.** *A*, averaged [Ca^2+^]_mit_ recordings from naïve, MCU-depleted, and MCU-overexpressing cells exposed to 30 min of OGD. *Traces* are the averaged [Ca^2+^]_cyt_ responses measured in the indicated conditions. *B*, [Ca^2+^]_mit_ levels recorded before and 30 min after the initiation of OGD under the different conditions. Data are mean ± S.E. from three to six recordings. The total number of measured cells is indicated inside the *bars. C*, effect of MCU silencing and overexpression on LDH release. Data are mean ± S.E. *n* is indicated inside the *bars. D*, Cav3.2 immunostaining in a cell expressing a matrix-targeted RFP. A significant fraction (56%) of the Cav3.2 immunoreactivity colocalized with the RFP fluorescence (*white pixels*). *E*, Western blot analysis showing the monomeric (*mon*) and dimeric (*dim*) forms of the MCU protein, which increased upon MCU overexpression (*MCU*) and decreased upon MCU silencing (*shMCU*) in a mitochondrial-enriched preparation. The insets show blot quantification. *C*, control. *F*, [Ca^2+^]_cyt_ levels recorded before and 30 min after the initiation of OGD in naïve and MCU-overexpressing cells. Data are mean ± S.E. from three to six independent recordings. The total number of measured cells is indicated inside the *bars. ns*, non-significant; *, *p* < 0.05; **, *p* < 0.01; ***, *p* < 0.001 (Student's *t* test for unpaired samples).

**FIGURE 6.**
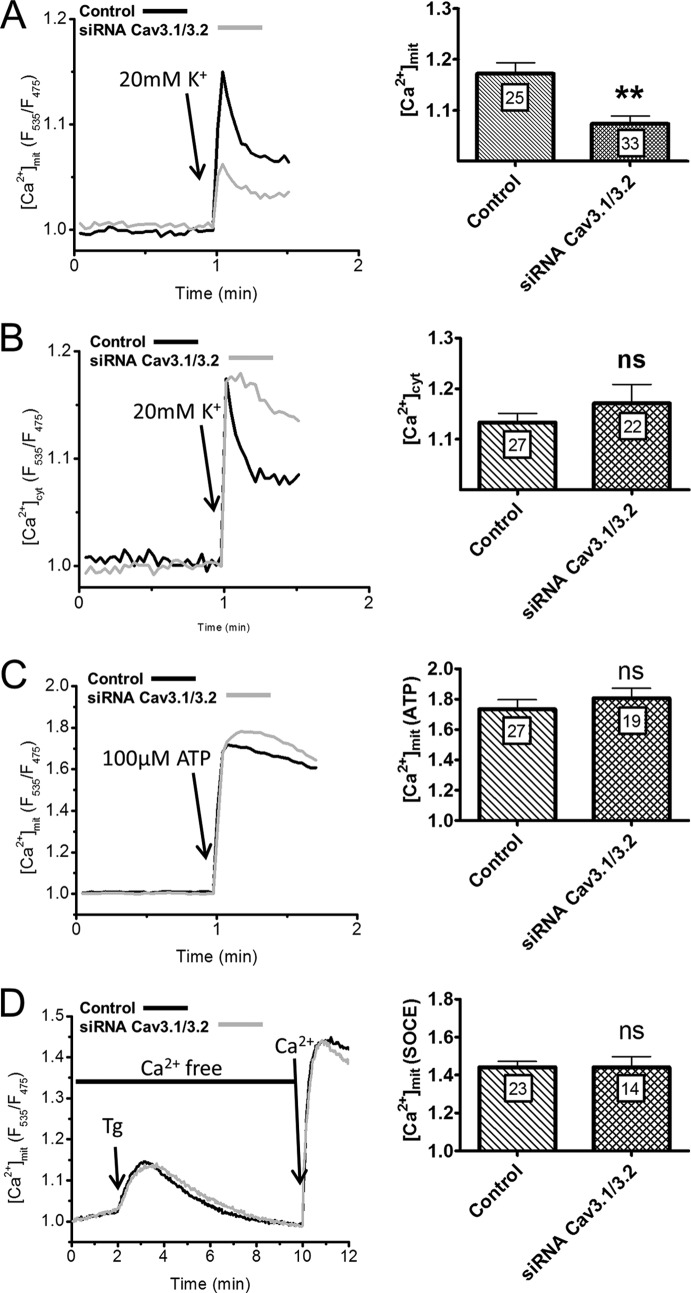
**Effect of T-type Ca^2+^ channel activation on [Ca^2+^]_mit_ and [Ca^2+^]_cyt_.** *A*, [Ca^2+^]_mit_ responses evoked by the addition of 20 mm K^+^ to naïve and Cav3.1/Cav3.2-depleted cells. Cav3.1/Cav3.2 silencing significantly decreased the amplitude of the [Ca^2+^]_mit_ response. *B*, [Ca^2+^]_cyt_ responses recorded under the same conditions. *C*, [Ca^2+^]_mit_ responses evoked by the addition of 100 μm ATP to evoke rapid Ca^2+^ release from intracellular stores. *D*, [Ca^2+^]_mit_ responses evoked by the addition of thapsigargin followed by Ca^2+^ readmission to promote Ca^2+^ entry across store-operated Ca^2+^ entry channels. Cav3.1/Cav3.2 silencing had no effect on the mitochondrial Ca^2+^ responses associated with ER Ca^2+^ release or store-operated Ca^2+^ entry-mediated Ca^2+^ influx. Data are mean ± S.E. from three to six independent recordings. The total number of measured cells is indicated inside the *bars. ns*, non-significant; **, *p* < 0.01 (Student's *t* test for unpaired samples).

To test whether the [Ca^2+^]_mit_ elevations contributed to OGD toxicity, we altered the expression levels of the major Ca^2+^ uptake protein of mitochondria, the mitochondrial Ca^2+^ uniporter (MCU) (16, 17). MCU overexpression, validated by Western blot (Fig. 5*E*), increased the delayed phase of the [Ca^2+^]_mit_ elevation evoked by OGD as well as resting [Ca^2+^]_mit_ levels (Fig. 5*A* and *B*) without altering the [Ca^2+^]_cyt_ elevations evoked by OGD (Fig. 5*E*) Conversely, MCU silencing, validated by Western blot (Fig. 5*E*), decreased the [Ca^2+^]_mit_ elevation evoked by OGD but did not alter basal [Ca^2+^]_mit_ levels (Fig. 5*A* and *B*). The increased [Ca^2+^]_mit_ elevation of MCU overexpressers was prevented by Ni^2+^, suggesting that a fraction of the Ca^2+^ taken up by mitochondria originated from T-type Ca^2+^ channels. Consistent with a role for mitochondrial Ca^2+^ uptake in cytotoxicity, MCU overexpression increased whereas MCU silencing decreased the cytotoxicity evoked by OGD (Fig. 5*C*). The deleterious effect of MCU expression was also prevented by Ni^2+^ (Fig. 5*C*). The opposite effects of Ni^2+^ and of MCU overexpression on [Ca^2+^]_mit_ and cytotoxicity suggests that mitochondria might be located close to T-type Ca^2+^ channels. Accordingly, expression of a matrix-targeted RFP revealed a high density of mitochondria in PC12 cells, with numerous mitochondria located beneath the plasma membrane that co-localized extensively with Cav3.2 immunoreactivity (Fig. 5*D*, *Mander's coefficient*, 0.56, *n* = 18). This indicates that the majority of Cav3.2 channels were located in close proximity to subplasmalemmal mitochondria. To test whether Ca^2+^ ions entering across T-type Ca^2+^ channels reach mitochondria, we used a low potassium concentration (20 mm) to depolarize the plasma membrane to ∼-50 mV and selectively activate T-type Ca^2+^ channels. This protocol evoked rapid increases in [Ca^2+^]_mit_ that were reduced by Cav3.1/Cav 3.2 silencing (Fig. 6*A*). The mitochondrial responses evoked by ER Ca^2+^ release or by SOCE channel activation were not affected by Cav3.1/Cav 3.2 silencing (Fig. 6*C* and 6*D*), validating the specificity of the siRNA. These data indicate that Ca^2+^ ions entering across T-type Ca^2+^ channels are taken up by mitochondria. These mitochondria must be located close to the influx channels as mitochondrial Ca^2+^ uptake only occurs at microdomains of high Ca^2+^ concentration (27). Accordingly, the [Ca^2+^]_cyt_ elevations evoked by 20 mm K^+^ were not affected by Cav3.1/Cav 3.2 silencing (Fig. 6*B*), indicating that incoming Ca^2+^ ions are preferentially transmitted from T-type Ca^2+^ channels to mitochondria. These data indicate that Cav3.2 channels are functionally coupled to subplasmalemmal mitochondria and contribute to pathological [Ca^2+^]_mit_ elevations during OGD.

## DISCUSSION

We report here that T-type Ca^2+^ channels contribute to cytotoxic Ca^2+^ elevations occurring during ischemia in PC12 cells, and that this toxicity is mediated at least in part by mitochondrial Ca^2+^ uptake. Consistent with earlier reports (31), we found that PC12 cells express very little amounts of T-type channels. Although we detected mRNA for both Cav3.1 and Cav3.2 by quantitative PCR, we could only document expression of Cav3.2 channels by immunofluorescence, and only a small fraction of cells was decorated with the Cav3.2 antibody. Moreover, we could not record endogenous T-type Ca^2+^ currents in PC12 cells, even with the sensitive tail current protocol described in (31), whereas we could readily record T-type Ca^2+^ currents when overexpressing Cav3.2 channels. Although we cannot rule out that T-type Ca^2+^ channels are activated or recruited during OGD as we could not record cells under these conditions, these data indicate that native T-type Ca^2+^ channels are expressed at very low level at the PM of PC12 cells. Despite their low abundance, we could obtain functional evidence of T-type Ca^2+^ channel activity by recording Ca^2+^ elevations evoked by small membrane depolarization steps with low concentrations of K^+^. More importantly, we observed that T-type Ca^2+^ channels contributed significantly to the cytotoxicity associated with OGD, and could relate this protective effect to specific alterations of cellular Ca^2+^ signals. Our long-term recordings with genetically encoded indicators revealed that [Ca^2+^]_cyt_ levels increase steadily for 2 h during OGD. External Ca^2+^ removal nearly abrogated this sustained [Ca^2+^]_cyt_ elevation and decreased OGD cytotoxicity, confirming that Ca^2+^ influx contributes to ischemia-induced Ca^2+^ overload and cell death. Remarkably, T-type channel knock-down or inhibition with low doses of Ni^2+^ decreased OGD cytotoxicity to levels similar than Ca^2+^ removal, but had much more specific effects on [Ca^2+^]_cyt_ that were restricted to the delayed phase of the sustained [Ca^2+^]_cyt_ elevation. The main effect of T-type channel inhibition was to slow the rates of the secondary [Ca^2+^]_cyt_ increase, without impacting on the amplitude of the first rapid elevation. This suggests that T-type Ca^2+^ channels are activated with a delay following OGD and contribute only a fraction of the Ca^2+^ influx-dependent [Ca^2+^]_cyt_ elevation during anoxia. T-type channel inhibition also delayed the decline in cellular ATP levels during OGD, likely by decreasing the Ca^2+^ load that must be actively extruded by plasma membrane Ca^2+^ ATPase. In contrast, overexpression Cav3.2 channels potentiated the sustained phase of the [Ca^2+^]_cyt_ response and maximally increased OGD toxicity. Cav3.2 overexpression also increased basal [Ca^2+^]_cyt_ levels, indicating that enforced expression of T-type Ca^2+^ channels might disrupt cellular Ca^2+^ homeostasis. These data link the protective effect of T-channels inhibitors reported previously in rat hippocampal neurons (11, 12) to decreases in pathological Ca^2+^ elevations. More importantly, the observation that manipulations of T-type channel expression preferentially alter the delayed Ca^2+^ signals during OGD yet maximally modulate the ensuing cytotoxicity suggests that the Ca^2+^ ions entering across these channels are particularly toxic.

Our results indicate that Ca^2+^ transfer to subplasmalemmal mitochondria might explain the unexpected toxicity of Ca^2+^ ions entering across T-type channels during ischemia. Sustained [Ca^2+^]_mit_ elevations can trigger the release of pro-apoptotic factors that initiate cell death (35, 36). We show here that mitochondria are exposed to large Ca^2+^ loads during ischemia and demonstrate the toxicity of mitochondrial Ca^2+^ uptake by showing that cells overexpressing the MCU are more sensitive to ischemia-induced cell death, whereas cells depleted of MCU are protected. MCU overexpression was shown to be pro-apoptotic (17), but whether increased mitochondrial Ca^2+^ uptake contributes to acute toxicity during ischemia is not known. We show that MCU overexpression increases mitochondrial [Ca^2+^] during the ischemic insult, whereas MCU depletion has the converse effect, demonstrating that a Ca^2+^ increase restricted to mitochondria is sufficient to potentiate cell death. It should be noted, however, that we did not explore the impact of T-type channels and MCU manipulations on [Ca^2+^]_cyt_ and [Ca^2+^]_mit_ during reperfusion. We observed that, for OGD lasting up to 90 min, [Ca^2+^]_cyt_ levels rapidly returned to basal levels upon reperfusion. However, for longer periods of OGD, we observed instead that [Ca^2+^]_cyt_ did not regain resting levels but remained elevated for several minutes (Fig. 1*A*). Further experiments are needed to test whether Cav3.2 channels and the MCU also contribute to [Ca^2+^]_cyt_ and [Ca^2+^]_mit_ elevations during this recovery phase. Importantly, the [Ca^2+^]_mit_ elevations evoked by OGD were increased by Cav3.2 overexpression and decreased by their invalidation or pharmacological inhibition, indicating that mitochondria take up Ca^2+^ ions flowing across T-type channels.

Subplasmalemmal mitochondria were shown previously to take up Ca^2+^ ions entering across voltage-gated Ca^2+^ channels in chromaffin cells, an uptake facilitated by the generation of high Ca^2+^ microdomains around the large capacity L-type channels (26). In contrast, high Ca^2+^ microdomains were not detected around mitochondria during the opening of store-operated Ca^2+^ entry channels, a finding that was explained by the exclusion of mitochondria from the contact sites between the ER and the PM where SOCE channels activate (27). Here, we show that a fraction of subplasmalemmal mitochondria is located in proximity to T-type Ca^2+^ channels, whose activation does not require the apposition of the ER. However, T-type Ca^2+^ currents are of much lower amplitude and duration than L-type Ca^2+^ currents, and, as discussed above, the density of T-type channels in PC12 cells is very low. The observation that mitochondria can take up the Ca^2+^ entering across T-type channels therefore points to a privileged communication between mitochondria and these Ca^2+^ channels. We could obtain evidence for this functional coupling by recording [Ca^2+^]_mit_ increases evoked by membrane depolarization to voltages (∼-50 mV) that selectively activate T-type Ca^2+^ channels. Efficient Ca^2+^ transfer from PM to mitochondria is also suggested by our observation that Ni^2+^ specifically reduced resting [Ca^2+^]_mit_ and not [Ca^2+^]_cyt_ levels. Ni^2+^ also inhibits Ca^2+^ uptake by isolated mitochondria (37), but such an inhibition would require millimolar concentrations in intact cells because of the low affinity of the divalent metal transporter 1 (38, 39). Our data therefore indicate that facilitated Ca^2+^ transfer from T-type Ca^2+^ channels to subplasmalemmal mitochondria contributes significantly to the toxicity during ischemia.

## Footnotes

2 The abbreviations used are:

[Ca^2+^]_cyto_: cytosolic [Ca^2+^]
[Ca^2+^]_mito_: mitochondrial matrix [Ca^2+^]
ER: endoplasmic reticulum
PM: plasma membrane
MCU: mitochondrial Ca^2+^ uniporter
OGD: oxygen and glucose deprivation
LDH: lactate dehydrogenase.
